# Public attitude towards quarantine during the COVID-19 outbreak

**DOI:** 10.1017/S0950268820002204

**Published:** 2020-09-21

**Authors:** W. Song, F. J. Sawafta, B. M. Ebrahem, M. A. Jebril

**Affiliations:** 1 Department of Psychology, Wuhan Sports University, China Resources, Hubei, 4300079, China; 2 Department of Immunology, Wuhan Sports University, Wuhan 4300079, China; 3 Department of Epidemiology and Health, Xian Jiaotong University, Xian 710049, China

**Keywords:** COVID-19, public attitudes, quarantine

## Abstract

Due to the outbreak of the deadly coronavirus disease in 2019 (COVID-19), Wuhan was on lockdown for more than 60 days by the state government. This study investigated the perceptions and attitudes of the public on quarantine as a practical approach to halting the spread of COVID-19. An online survey was conducted via WeChat between 10 January 2020 and 10 March 2020 on the general population in Hubei province at the height of the COVID-19 outbreak. In total, 549 respondents participated in the survey. Results revealed that the public displayed significantly strong support towards quarantine throughout the outbreak period, apart from locking people up and using imprisonment legal sanctions against those who failed to comply with the stringent regulations. The support exerted by the public stemmed from the execution of authorised officers to protect the public interest and provision of psychosocial support for those affected. In situations where quarantine could not be imposed, public health policy-makers and government officials should implement an extensive system of psychosocial support to safeguard, instruct and inform frontline public health workers. The public should also be enlisted in an open conversation concerning the ethical utility of restrictive values during the COVID-19 outbreak.

## Introduction

Quarantine has become a significant approach in combatting the coronavirus disease in 2019 (COVID-19), which has spread to all major cities in China. Thousands of people and foreigners who are residing in China or have returned home from China have been affected by this disease, causing a worldwide pandemic [1]. The ‘quarantine’ approach is referred to as the process of separating and restricting the movement of people to halt the spread of contagious disease [2]. The term has been initially coined by society in 1127 to curb leprosy that had spread throughout Italy (Venice). Quarantine has since been used as a term across the world to describe movement control of the people when an epidemic happens. The west African countries have employed quarantine during the 2014 Ebola outbreak. Similarly, the UK, Canada and Beijing have also responded to the severe acute respiratory syndrome (SARS) outbreak that is caused by a coronavirus in 2003 through quarantine [3]. Quarantine differs from isolation as the latter reflects separating those diagnosed with a disease from the rest of the population who are healthy. However, these terms have been interchangeably used when addressing the general public throughout the COVID-19 outbreak [4].

COVID-19 is currently assumed to have begun on 23 December 2019 when an emerging cluster linked to the Hunan Seafood Wet Market in Wuhan caused many people to be infected with pneumonia symptoms without any apparent cause. Nonetheless, Chinese scientists are subsequently able to link pneumonia to a new strain of coronavirus called COVID-19 [5]. The virus spread within the Hubei province before infecting all provinces in China, and more than 50 other countries worldwide by 14 March 2020. Since the virus first spread in Wuhan and has been the largest outbreak of COVID-19 within the community at that time, there is considerable panic in the Wuhan city due to the rapid transmission and mortality rate. Hence, quarantine has been enforced, apart from executing other effective measures, due to several reasons. Quarantine would restrict the spread of COVID-19, reduce the peak activity of the virus in the community and minimise the death rate in the absence of a vaccine or effective treatment [6, 7]. Thus, all transportation services in the Wuhan city are shut down by the central government of China, which would further control the movement of the residents [8]. Both symptomatic and asymptomatic individuals are told to isolate themselves as the virus has a 14-day incubation period when transmitted from a human to another [9–12].

Despite the long history of quarantine implemented worldwide, the perceptions and attitude of the general public towards quarantine are untapped, especially in this modern era. The measures taken during quarantine in Wuhan has left many people with an unpleasant experience, such as losing contact with loved ones, sacrificing freedom, breeding fear and being stuck in boredom [13]. Some individuals have also argued the ineffectiveness of executing quarantine to halt the spread of COVID-19 [14, 15]. Besides, three previous analyses have stated that a lengthy quarantine period could be associated with frustration, deteriorating mental health, fear, anxiety, stigma, stress, uncertainty and depression. Based on the adverse effects of psychological and physical health from quarantine, there is, therefore, a need to investigate the perceptions and attitudes of the public on quarantine amid COVID-19 outbreak. Specifically, this study intends to assess the perceptions and attitudes of the people in Hubei province towards quarantine as a strategic approach to curb the spread of COVID-19 outbreak [16, 17].

## Methods

This study was conducted in the Hubei province of Central China, which included the first epicentre of the COVID-19 outbreak and capital of the province, Wuhan. The public health authority had locked down the entire Hubei province and restricted the movement of the people. Besides, individuals who had contact with COVID-19 patients were quarantined in a separate room at home. As a result, the social network known as WeChat became the most widely used application in China that incorporated the daily lives of the people throughout the quarantine period [18, 19]. Thus, this study had incorporated an online survey through WeChat in collecting data, which also focused on a one-response-one-person approach.

The potential respondents were screened based on specific criteria within this study before receiving a WeChat code. The inclusion criteria in selecting the survey respondents include being at the minimum age of 18 years, having good Chinese comprehension skill, as well as residing within the Hubei province as the primary residence throughout the COVID-19 outbreak. Respondents also needed to give consent to participate in the survey. A total of 549 individuals who met the inclusion criteria completed the survey instrument.

The survey instrument was designed based on the Chinese Public Health policy in understanding the perceptions and attitudes of the general public towards quarantine as an approach to stop the spread of COVID-19. A pre-test was conducted on the survey to ensure the length, and the objectives were met. The items embedded in the questionnaire were: restrictive measures on legality perceived efficiency of quarantine and expected support provided to those being quarantined. The respondents were required to indicate the level of agreement or disagreement for each question based on a rating scale of 1 to 5 (1 = strongly agree, 2 = somewhat agree, 3 = neutral, 4 = somewhat disagree and 5 = strongly disagree) (see the Appendix).

The following standardised definition of quarantine was provided at the beginning of the survey:

‘Quarantine means you should stay in a separate area away from others because you have been around someone with a new type of viral infectious disease and you may have it, too’.

Respondents were required to describe individuals who broke the quarantine rules by choosing one of the following: (1) driving without wearing a seatbelt, (2) driving under the influence of alcohol or intoxicating substance and (3) physical assault. Before data collection, approval of the research ethics was obtained from Wuhan Sports University and Hubei Public Health. The data collection process commenced from 10 January to 10 March 2020.

The multivariate and bivariate analyses were used to assess the interrelationships among the variables. These statistical analyses were carried out using the SPSS 12.0 software package for Windows. The sample size was, nonetheless, not calculated but estimated in the form of convenient sampling that was based on the availability of the respondents. Thus, the sample was not representative of the population in the Hubei province due to the inconvenience during data collection. On the other hand, Varimax rotation and Kaiser normalisation were employed to simplify the expression of a particular sub-space of a few critical items. Each of these items was calculated based on the factor analysis tools in SPSS, dimension reduction of factor and principal components of the eigenvalues that should be greater than 1 [20].

## Results

A total of 620 participants had completed the online questionnaire. However, only 549 respondents were included in the final analysis after excluding 71 questionnaires with incomplete responses (response rate: 88.5%). The demographic characteristics of the respondents are tabulated in Table 1. About 52.8% of the respondents were female, with 72% were between 18 and 35 years old, 25% were between 36 and 55 years old and 3% were 55 years and above. From these 549 respondents, only 49.1% were residing in Wuhan city, with 60.47% had undergraduate university or college qualification and 52.27% earned an average monthly income of less than 5000 Yuan. A total of 9% of the respondents were personally affected by quarantine due to the COVID-19 outbreak.

**Table 1.** Demographic characteristics of the participants

Individual characteristics	Female	Male	Total
Age group			
18–35 years	212	185	397
36–55 years	70	65	135
>55 years	8	9	17
Reign			
Wuhan city	123	147	270
Hubei province except for Wuhan city	183	96	279
Education			
High school or less	25	41	66
Undergraduate university or college	190	142	332
Postgraduate	75	76	151
Income			
<5000 RMB	140	147	287
⩾5000 RMB	150	112	262
Anyone in your family quarantined during the COVID-19			
No	263	236	499
Me but nobody else at home quarantined	10	5	15
Me and the rest of my family	13	14	27
Not me but the rest of my family	4	4	8

Table 2 shows the results of the 15 items (ranged from ‘Strongly Agree’ to ‘Strongly Disagree’) in the survey. Although these items were individually probed in the survey, the items were clustered based on the outcomes from the factor analysis for better presentation. Variance that was insignificant was distinguished for respondents between Wuhan city and Hubei province (without Wuhan city). Results showed that most of the respondents responded either ‘strongly agree’ or ‘somewhat agree’ to each of the items, which suggested the presence of self-sufficient vindication for quarantine throughout the COVID-19 outbreak. A majority of the respondents claimed that health officials should reduce the burden endured by those under quarantine. Most of the respondents believe that the execution of compulsory measures during quarantine was necessary to protect the public. Based on the results, 92% of the respondents believed that legal penalties and punishments should be enforced to those who refused to comply with the regulations. This result highlighted the majority response on the importance of quarantine to protect family, friends and community from the disease. Although these high proportions of agreement on the justification of quarantine suggested a certain degree of convergence in public opinion, further investigation using Xems on both the responses for ‘Strongly Agree’ and ‘Somewhat Agree’ varied significantly across the items. The results are displayed in Table 2.

**Table 2.** Public attitudes towards quarantine during the COVID-19 outbreak

Items	Strongly agree (%)	Somewhat agree (%)	Natural (%)	Somewhat disagree (%)	Strongly disagree (%)
Justification					
The public health department has the authority on people during an outbreak	72	16	9	2	1
Quarantine is the best way to stop the spread COVID-19	79	13	7	1	0
If someone is informed by the public health department on the need to be quarantined, the individual should abide by the instruction	52	22	19	5	2
Quarantine guarantees my family, friends, community and I to be protected from being infected	62	20	12	4	2
Sanctions					
People who break the quarantine regulations should face fines or imprisonment	75	17	6	1	1
The public health department has the right to imprison an individual who does not follow the quarantine instruction	58	22	14	5	1
The public health department should use electronic bracelets and home surveillance cameras on those who do not comply with quarantine instructions	24	23	24	20	9
Burdens					
Public health should explain the need for quarantine to the public	63	20	12	3	2
The government should pay for medical staff and volunteers to help people who are in quarantine	60	23	15	1	1
The government should ensure food and shelter for people during quarantine and use public funds if necessary	60	22	15	2	2
The government should pay consultants and support groups to manage people affected by quarantine	34	30	29	5	2
People in quarantine should receive compensation from the government in replace of salary and wages	21	17	37	20	5
Safeguards					
The public health departments should ensure that there is no discrimination during a quarantine	75	11	11	2	1
Some human rights are bound to be deprived during the COVID-19 outbreak	29	32	25	9	5
People who are not in favour of quarantine should provide feedback to the authority to end the quarantine as soon as possible	27	26	19	12	16

Some respondents (13%) answered that quarantine is most likely driving without wearing a seatbelt that can allow a police officer to issue a ticket for breaking the quarantine rule, 22% selected driving under the influence of alcohol or intoxicating substance as a large risk factor that contributes to traffic collisions, 2% failed to provide response and 63% linked quarantine with physical assault or a crime.

The variance analysis displayed statistical significance for sex and age. The score for justification sub-scale among female respondents was significantly higher than males (*F* = 3.25 (df = 4), *P* < 0.05). This result exhibited a strong agreement in justifying the quarantine approach during COVID-19 outbreak. Besides, respondents aged between 36 and 55 years old showed stronger agreement that the execution of quarantine was justified than both younger and older respondents (>65 years) (*F* = 3.46 (df = 4), *P* < 0.05). On the other hand, the younger respondents agreed most strongly that penalties for those who defied quarantine as appropriate (*F* = 3.46 (df = 4), *P* < 0.05). Comparatively, respondents from the middle-aged group (between 36 and 55 years old) only agreed more strongly that punishments for people who flouted quarantine as appropriate (*F* = 3.57 (df = 4), *P* < 0.05). Furthermore, the safeguards sub-scale for respondents from the middle-aged group (between 36 and 55 years old) scored significantly higher than the younger and older respondents (*F* = 4.20 (df = 4), *P* < 0.05). This result indicated the strong agreement within the middle aged group that quarantine did safeguard the community during the COVID-19 outbreak. However, there was no significant difference noted for education level, household income and region (see Table 3).

**Table 3.** Analysis of variance testing for public attitudes towards quarantine, which branched under four structure sub-scales that include: Justification’, ‘Sanctions’, ‘Burdens’ and ‘Safeguards’

Variables	Justification			Sanctions			Burdens			Safeguards		
	*F* value	df	*P* value	*F* value	df	*P* value	*F* value	df	*P* value	*F* value	df	*P* value
Age (years)												
18–35	4.50	4	0.017	3.46	4	0.041	3.81	4	0.060	3.99	4	0.047
36–55	3.46	4	0.002	3.57	4	0.045	4.05	4	0.063	4.20	4	0.003
>55	5.14	4	0.103	4.24	4	0.120	3.28	4	0.094	3.58	4	0.450
Gender												
Male	1.98	4	0.072	0.97	4	0.099	0.87	4	0.075	0.87	4	0.071
Female	3.25	4	0.003	3.14	4	0.067	2.11	4	0.081	2.36	4	0.065
Region												
Wuhan citizen	5.54	4	0.098	4.17	4	0.107	5.11	4	0.101	5.18	4	0.081
Foreigners residing in Wuhan	4.68	4	0.151	4.88	4	0.122	4.21	4	0.188	4.21	4	0.114
Education level												
High school or less	1.48	4	0.315	2.44	4	0.125	1.54	4	0.216	1.88	4	0.327
Undergraduate university or collage	1.81	4	0.183	1.24	4	0.089	1.57	4	0.116	1.92	4	0.124
Postgraduate	1.99	4	0.063	2.10	4	0.120	2.06	4	0.077	1.74	4	0.087
Income												
<5000 RMB	2.37	4	0.111	3.01	4	0.198	2.87	4	0.122	2.74	4	0.143
⩾5000 RMB	3.54	4	0.122	3.55	4	0.107	3.57	4	0.178	3.11	4	0.110

## Discussion

Quarantine is an intricate, ethical and legal form of intervention in ensuring the safety and sanity of the people during an epidemic, or in this study, a global pandemic [21]. Results from this study have revealed that many respondents consider quarantine as a crucial method to put a stop to the contagious COVID-19. Many respondents have also shown strong support towards execution of quarantine, penalties that are imposed on people who disobeyed the rules during quarantine, social support for those under quarantine and public protection.

Statistical results from previous studies have reported that societal norms and culture can affect quarantine compliance levels. People are generally found to prefer quarantine on those suspected of or suffering from infectious disease based on the following statistics: 95% in Taiwan, 76% in the USA and 89% in Singapore [22]. These results support findings from this study, whereby 92% of the respondents have agreed that quarantine is the best way to stop the COVID-19 outbreak. Besides, 82% agreed that quarantine is the best way to protect family, friends and community from being infected.

This study has also reported that 92% of the respondents agreed that those who broke the quarantine regulations should face fines or imprisonment legal sanctions. Moreover, 80% have agreed that the public health department has the right to imprison those who do not adhere to the quarantine order. However, a study from the Harvard University on people who have been quarantined due to SARS show that proportions who agree to quarantine decrease significantly (42% in the USA and 70% in Taiwan) if people could be detained for refused quarantine orders. The researchers have attributed the difference to the experience with contagious disease outbreaks, whereby quarantine and other restrictive values have been executed [23].

Public health at the global scale has also appeared to be in a state of confusion regarding the implementation of quarantine and other drawbacks of the outbreak that affect human rights. Some consider quarantine policy during outbreaks of contagious diseases as a judicious health policy [24], while others consider quarantine as an unneeded breach of human rights [25]. However, public health authorities must rely on persuasion to provide sufficient and clear guidelines about the purpose of quarantine [23, 26, 27]. However, during the outbreak of COVID-19, quarantine significantly has reduced the spread of infectious disease. This survey has also revealed that quarantine is an obligation on the public health community that describe both the restriction to human rights and obligation of the public health regime within the COVID-19 outbreak context.

There are several limitations to this study. First, the data are limited only to Hubei province. Thus, future studies should be carried out in other provinces and countries to compare the results. This study has been conducted during the COVID-19 outbreak, so public perceptions and attitude in the first 14 days or after the outbreak could differ. Besides, a majority of the respondents have been young adults (18–35 years old). Hence, more senior residents are under-represented. Future studies may focus on senior residents via face-to-face interviews, which could not be used during this newly emerging infectious disease outbreak. Data on perceptions and attitude towards quarantine have been self-reported in this study. Thus, the validity of the data is limited.

## Conclusion

In the early stages of this newly emerging infectious disease, authorities have little information about the outbreak. With only data that have shown rapid transmission through an unknown causative agent, duration of communicability, mode of transmission and incubation period, the virus has been a threat to the population at that time. Thus, quarantine is the most suitable method to be used to protect the public by preventing those who have or may have this infection from getting into contact with society at large. However, such a restrictive measure requires balance and justification between individual rights and community rights. The current findings indicate that many people agree to quarantine as a necessary method in managing COVID-19. There should also be severe punishments for those who flout quarantine through public support to safeguard the society from irresponsible individuals. Psychosocial support should also be made available to provide for individuals who are affected by the outbreak. This intensity between individual rights for the greater good of the society and public health morals have been the challenge of infectious disease. Therefore, the authorities and public health policy-makers should execute an extensive system of support and generate proper public support for the necessary measures for quarantine and other restrictive values. These measures would have instructions to inform the frontline public health workers, as well as to enlist the public at large in an open conversation, on the ethical utility of restrictive values during the COVID-19 outbreak.

## Data Availability

The data that support the findings of this study can be downloaded from the Zenodo at DOI: d10.5281/zenodo.4034292.

## Supplementary material

For supplementary material accompanying this paper visit http://dx.doi.org/10.1017/S0950268820002204. click here to view supplementary material

## Appendix

Respondents were asked to score their responses to the following questions on a scale of 1–5 where:

1 = Strongly agree
2 = Somewhat agree
3 = Neutral
4 = Somewhat disagree
5 = Strongly disagree

Below is a list of 15 items to measure public attitudes and perceptions towards the usage of quarantine during COVID-19 outbreak. Please read each item, and then indicate your level of agreement/disagreement with each question

1. The public health department has the authority on people during an outbreak.
2. Quarantine is the best way to stop the spread COVID-19.
3. If someone is informed by the public health department on the need to be quarantined, the individual should abide by the instruction.
4. Quarantine guarantees my family, friends, community and I to be protected from being infected.
5. People who break the quarantine regulations should face fines or imprisonment.
6. The public health department has the right to imprison an individual who does not follow the quarantine instruction.
7. The public health department should use electronic bracelets and home surveillance cameras on those who do not comply with quarantine instructions.
8. Public Health should explain the need for quarantine to the public.
9. The government should pay for medical staff and volunteers to help people who are in quarantine.
10. The government should ensure food and shelter for people during quarantine and use public funds if necessary.
11. The government should pay consultants and support groups to manage people affected by quarantine.
12. People in quarantine should receive compensation from the government in replace of salary and wages.
13. The public health departments should ensure that there is no discrimination during a quarantine.
14. Some human rights are bound to be deprived during the COVID-19 outbreak.
15. People who are not in favour of quarantine should provide feedback to the authority to end the quarantine as soon as possible.
