# Supplementary material for: Public attitude towards quarantine during the COVID-19 outbreak
Source: Epidemiol Infect. 2020 Sep 21;148:e220. doi: 10.1017/S0950268820002204 (PMC7533476; doi:10.1017/S0950268820002204)
Supplement: Supplementary file 1 [file S0950268820002204sup001.docx]

Table 2

Public attitudes toward quarantine during the COVID-19 outbreak. For 9 % of respondents were people affected by quarantine ordered to stay home in a separate room

| Items | Strongly Agree | Somewhat Agree | Natural | Somewhat Disagree | Strongly Disagree |
| --- | --- | --- | --- | --- | --- |
| Justification  The public health department has authority to direct people during outbreaks  Quarantine is the best way to stop the spread covi-19 outbreak.  If someone is informed by the public health department that they need to be quarantined, they should follow no matter what changes they will bring to their lives.  If I am guaranteed my family, friends, and community will be protected from being infected. | 72%  79%  52%  62% | 16%  13%  22%  20% | 9%  7%  19%  12% | 2%  1%  5%  4% | 1%  0%  2%  2% |
| Sanctions  People who break the quarantine regulations should face fines or imprisonment legal sanctions.  If the people do not follow the quarantine order, the public health department has the right to lock them up  The public health department should respond to the use of electronic bracelets and home surveillance cameras by those who do not comply with quarantine orders | 75%  58%  24% | 17%  22%  23% | 6%  14%  24% | 1%  5%  20% | 1%  1%  9% |
| Burdens  Public Health should explain to everyone why they are being quarantined.  The government should pay for medical staff and volunteers to help people who are in quarantine.  The government should ensure food and shelter for people during the quarantine period and paid for with public funds if necessary  The government should pay consultants and support groups so that people who have been removed from quarantine have someone to talk to about it  People in quarantine should receive compensation from the government for their work during the period of quarantine | 63%  60%  60%  34%  21% | 20%  23%  22%  30%  17% | 12%  15%  15%  29%  37% | 3%  1%  2%  5%  20% | 2%  1%  2%  2%  5% |
| Safeguards  The public health departments should ensure that there is no discrimination in the quarantine  During the outbreak of COVID-19, it is reasonable for some rights to be deprived.  People who not favor with their quarantine order should be capable of asking a review to have it ended soon as possible | 75%  29%  27% | 11%  32%  26% | 11%  25%  19% | 2%  9%  12% | 1%  5%  16% |

Table 2: Public attitudes toward quarantine during the COVID-19 outbreak. 91% who were not affected by quarantine but Public health authority restricted their movement for example stay inside the community, must wear mask

| Items | Strongly Agree | Somewhat Agree | Natural | Somewhat Disagree | Strongly disagree |
| --- | --- | --- | --- | --- | --- |
| **Justification**  The public health department has authority to direct people during outbreaks  Quarantine is the best way to stop the spread covi-19 outbreak.  If someone is informed by the public health department that they need to be quarantined, they should follow no matter what changes they will bring to their lives.  If I am guaranteed my family, friends, and community will be protected from being infected. | 64%  76%  54%  62% | 20%  13%  26%  22% | 12%  11%  11%  10% | 4%  0%  9%  6% | 0%  0%  0%  0% |
| **Sanctions**  People who break the quarantine regulations should face fines or imprisonment legal sanctions.  If the people do not follow the quarantine order, the public health department has the right to lock them up  The public health department should respond to the use of electronic bracelets and home surveillance cameras by those who do not comply with quarantine orders | 66%  60%  23% | 28%  22%  21% | 4%  14%  24% | 2%  4%  18% | 0%  0%  14% |
| **Burdens**  Public Health should explain to everyone why they are being quarantined.  The government should pay for medical staff and volunteers to help people who are in quarantine.  The government should ensure food and shelter for people during the quarantine period and paid for with public funds if necessary  The government should pay consultants and support groups so that people who have been removed from quarantine have someone to talk to about it  People in quarantine should receive compensation from the government for their work during the period of quarantine | 47%  56%  61%  37%  24% | 29%  22%  19%  27%  18% | 14%  22%  20%  29%  32% | 10%  0%  0%  7%  16% | 0%  0%  0%  0%  10% |
| **Safeguards**  The public health departments should ensure that there is no discrimination in the quarantine  During the outbreak of COVID-19, it is reasonable for some rights to be deprived.  People who not favor with their quarantine order should be capable of asking a review to have it ended soon as possible | 78%  35%  25% | 16%  30%  14% | 6%  17%  33% | 0%  10%  15% | 0%  8%  13% |
